# Incidence and Risk Factors for Neonatal Tetanus in Admissions to Kilifi County Hospital, Kenya

**DOI:** 10.1371/journal.pone.0122606

**Published:** 2015-04-07

**Authors:** Fredrick Ibinda, Evasius Bauni, Symon M. Kariuki, Greg Fegan, Joy Lewa, Monica Mwikamba, Mwanamvua Boga, Rachael Odhiambo, Kiponda Mwagandi, Anna C. Seale, James A. Berkley, Jeffrey R. Dorfman, Charles R. J. C. Newton

**Affiliations:** 1 KEMRI-Wellcome Trust Research Programme, Centre for Geographic Medicine Research (Coast), Kilifi, Kenya; 2 Centre for Tropical Medicine and Global Health, Nuffield Department of Clinical Medicine, University of Oxford, Oxford, United Kingdom; 3 International Centre for Genetic Engineering and Biotechnology, Cape Town, South Africa; 4 Division of Immunology, University of Cape Town, Cape Town, South Africa; 5 Department of Psychiatry, University of Oxford, Oxford, United Kingdom; University of Cambridge, UNITED KINGDOM

## Abstract

**Background:**

Neonatal Tetanus (NT) is a preventable cause of mortality and neurological sequelae that occurs at higher incidence in resource-poor countries, presumably because of low maternal immunisation rates and unhygienic cord care practices. We aimed to determine changes in the incidence of NT, characterize and investigate the associated risk factors and mortality in a prospective cohort study including all admissions over a 15-year period at a County hospital on the Kenyan coast, a region with relatively high historical NT rates within Kenya.

**Methods:**

We assessed all neonatal admissions to Kilifi County Hospital in Kenya (1999–2013) and identified cases of NT (standard clinical case definition) admitted during this time. Poisson regression was used to examine change in incidence of NT using accurate denominator data from an area of active demographic surveillance. Logistic regression was used to investigate the risk factors for NT and factors associated with mortality in NT amongst neonatal admissions. A subset of sera from mothers (n = 61) and neonates (n = 47) were tested for anti-tetanus antibodies.

**Results:**

There were 191 NT admissions, of whom 187 (98%) were home deliveries. Incidence of NT declined significantly (Incidence Rate Ratio: 0.85 (95% Confidence interval 0.81–0.89), P<0.001) but the case fatality (62%) did not change over the study period (P = 0.536). Younger infant age at admission (P = 0.001) was the only independent predictor of mortality. Compared to neonatal hospital admittee controls, the proportion of home births was higher among the cases. Sera tested for antitetanus antibodies showed most mothers (50/61, 82%) had undetectable levels of antitetanus antibodies, and most (8/9, 89%) mothers with detectable antibodies had a neonate without protective levels.

**Conclusions:**

Incidence of NT in Kilifi County has significantly reduced, with reductions following immunisation campaigns. Our results suggest immunisation efforts are effective if sustained and efforts should continue to expand coverage.

## Introduction

Neonatal tetanus (NT) continues to be a major cause of mortality and neurological sequelae for survivors yet it is highly preventable using simple and inexpensive public health interventions [1,2]. In 2013, NT was estimated to be responsible for 49,000 deaths [3], mostly in rural areas of developing countries where most births occur at home and are often attended by unskilled persons using unhygienic practices without aseptic postnatal care [4]. NT is estimated to contribute about 2% of neonatal deaths in 2012 [5], a decrease from 7% in 2000 [4], but has a very high case fatality rate [6,7].

Fetuses acquire passive immunity to tetanus if their mothers are adequately immunised. Two or more doses of tetanus toxoid vaccine to the mother have been shown to reduce NT mortality by 94% [8]. Immunisation may therefore reduce the number of NT cases to the World Health Organization (WHO) elimination target of ≤1 per 1000 live births in all regions, these targets have been missed twice (1995 and 2005) [9]. However, pregnant women may not get adequate immunisation because they cannot, or do not access antenatal care at all, or do so late in their pregnancy [10].

Studies have shown that NT deaths are underestimated with reporting proportions as low as 5% [11]. In 2000, Kenya was among the 59 countries having 11–50% of its districts at high risk of NT deaths [12]. Ten years later, NT was still a public health problem in 34 countries, including Kenya. Consequently, it was among the 10 countries selected by WHO to implement a policy of three doses of tetanus toxoid in high risk areas in the year 2012. By May 2013, Kenya was still among the 28 remaining countries yet to meet the elimination target [13].

Studies from Kenya have shown that NT has high case fatality and that those who survive have evidence of brain damage [14,15]. We aimed to determine changes in the incidence of NT, characterize and investigate the associated risk factors and mortality in a prospective cohort study including all admissions from 1999 to 2013 at a rural County hospital on the Kenyan coast.

## Methods

### Study setting and population

The study was conducted in Kilifi County Hospital (KCH) [formerly Kilifi District Hospital], the only County level hospital within the area covered by the Kilifi Health and Demographic Surveillance System (KHDSS) [16], in Kilifi County, Coastal region. The estimated fertility rate in KHDSS is 4.7 and the overall neonatal mortality rate was estimated at 17.1 per 1000 live births for the period 2006–2010 [16].

KCH serves the KHDSS population, with about 4400 paediatric admissions every year. KCH has one paediatric ward and a high dependency unit. The high dependency unit nurses triage all children, identifying those requiring immediate medical attention, including those with NT. Immunoglobulin and facilities for ventilation were unavailable during the period of the study.

### Diagnosis

The diagnosis of tetanus was clinical, based on medical history and examination, determining the presence of at least three of the following clinical findings: severe trismus, refusal to feed, generalised muscle rigidity, opisthotonus or spontaneous tetanic spasms [17]. A full clinical examination was performed on admission, with pulse and peripheral oxygen saturation measured by fingertip pulse oximetry (Nellcor). Respiratory rate was counted over 1 minute. The number of spasms was counted over a 5-minute period whilst the neonate lay on a bed in a side ward. A tetanus score based upon the summation of scores on: irritability (0–2), feeding problems (0–2), stiffness (0–3), frequency of spasms (0–3), duration of spasms (0–3), involvement of limbs (0–3), stimulation of spasms (0–3), cyanosis during spasms (0,-2) and apnoea (0–1) was calculated, with higher scores being indicative of more severe disease.

### Management

Care was primarily supportive, as tetanus immunoglobulin was not available, but the umbilical cord stump was cleaned every day with surgical spirit to reduce toxin production [18,19]. Broad spectrum antibiotics were given according to standard protocols; either intravenous pencillin (benzylpenicillin (50mg/kg 6 hourly) or ampicillin (50mg/kg 6 hourly)) with gentamycin (7.5mg/kg once daily IV) or metronidazole (7.5mg/kg 8 hourly).

Patients with NT were nursed in a quiet room on a bed covered with a cradle and blankets to protect the baby from light. During the period of severe spasms IV fluids were given at rate of 150–200mls/kg/day, but when these stopped breast milk was given through a nasogastric (ng) tube. Neonates were sedated with diazepam (0.1–0.25 mg/kg IV) and phenobarbital (15mg/kg loading dose ng, 5–8 mg/kg maintenance 12 hrly ng). Chlorpromazine (0.5 mg/kg/dose 6 hrly ng) was added if required due to persisting spasms. Oxygen was given when saturation dropped below 95% and suction was performed when necessary. With improvement, identified through daily monitoring and scoring of spasms, sedation was reduced and feeding recommenced prior to discharge.

### Investigations and laboratory methods for clinical investigations

Blood samples were taken within one hour of admission. Laboratory measurements included full blood count (Beckman Coulter Inc USA), electrolytes (Chriron Diagnositics 614 Na+/K+ analyser, Ciba Corning, UK), creatinine (Creatinine Analyzer 2, Beckman, USA) and glucose (Analox GM 6, London UK). Blood and cerebrospinal fluid (CSF) were taken for microscopy and bacterial culture (Bactec, BD).

### Measurement of antitetanus antibody levels by ELISA

Plasma was collected from blood drawn from the neonates with tetanus and their mothers and stored at -20°C for the measurement of IgG and IgG_1_ specific for tetanus toxoid using a standard enzyme-linked immunosorbent assay (ELISA). Briefly, tetanus toxoid protein (National Institute for Biological Standards and Control (NIBSC, Hertfordshire, England) was coated on standard 96 well ELISA plates and blocked with phosphate buffered saline with 0.05% Tween-20 (Sigma, Dorset, England) and 4% non-fat dry milk. Duplicate serial dilutions of each serum/plasma sample were then added to wells, incubated and then washed. Bound antibody to tetanus toxoid was detected with anti-human IgG or anti-human IgG_1_ conjugated to horseradish peroxidise (Dako, Glostrup, Denmark), followed by O-phenylenediamine (Sigma). Quantification was undertaken by comparison of values in the log-linear range to those of antitetanus antibody standard 26/388 [20] run in the same assay; standard was generously provided by Dr. D. House, Imperial College, London.

### Statistical analysis

Analyses were performed using STATA (Version 12; STATA Corp, College Station, TX, USA). Descriptive statistics were first undertaken and incidence and risk factors assessed as described below.

### Incidence of NT over time

We measured the incidence as the number of NT cases for every 1000 live births per year. Since we were not sure if the unregistered cases [neonates not already identified within the KHDSS system] came from within the KHDSS we considered both scenarios by treating them as either from within and outside the KHDSS. Cases that were explicitly stated to be from outside the KHDSS were not used in the calculation of incidence. However, unless it is explicitly stated otherwise, we refer to incidence as that arising from cases within the KHDSS. Data on live births in KHDSS formed the denominator, with reliable data on these taken from KHDSS for the period 2004 to 2012. We fitted a simple linear regression model to these data to predict the unknown number of live births for the period 1999 to 2003. Poisson regression was used to examine the secular trend of NT admissions with logarithm of the number of live births taken as an offset in the model.

### Factors associated with NT

Four hundred hospitalised neonatal controls admitted to KCH during the study period were randomly selected from the clinical database for the nested case-control study. Clinical data on place of birth, weight at admission and oxygen saturation at admission was obtained from routine clinical surveillance data. Multivariable logistic model was used to determine which of these factors were associated with admission with NT rather than other conditions. To identify the independent predictors, we first performed univariable associations and included all the variables with univariable P-value (P) <0.20 in the final multivariable model.

### Factors associated with death in NT admittees

For neonates residing within the KHDSS, survival data were obtained from the KHDSS. Yearly case fatality risk was calculated as the percentage of cases that died. Multivariable logistic regression was used to determine factors associated with death in NT by including all the explanatory variables with P<0.20 in the univariable analysis together in one model. Variables considered included: maternal factors (education of the mother and immunisation status), demographic factors (sex and age in days), clinical features (heart rate, respiratory rate, umbilicus (inflamed or not), prostration (present/absent), number of spasms, oxygen saturation (defined as low if below 95%) and presence or absence of complications during delivery, and laboratory investigations (hypoglycaemia defined as blood sugar <2.5mmol/L(<2.5kg), magnesium concentration, sodium level, creatinine level, white blood cell count and haemoglobin).

### Antitetanus antibodies

The plasma from 47 infants admitted to Kilifi County Hospital for tetanus between May 1999 and September 2003 and from 61 mothers were tested for IgG antibodies specific for tetanus toxoid (anti-TT IgG). All infant samples that were tested had a matching maternal sample. We thus tested maternal samples with detectable antitetanus antibody for antitetanus IgG_1_. We did not have permission to test for HIV or examine their placenta during this period.

### Ethics Statement

We obtained informed written consent from the neonates’ parent/guardian. The study was approved by the Kenya Medical Research Institute (KEMRI) and the KEMRI National Ethics Review Committee (SCC Number: 1592).

## Results

### Incidence of NT over time

**Fig 1:**
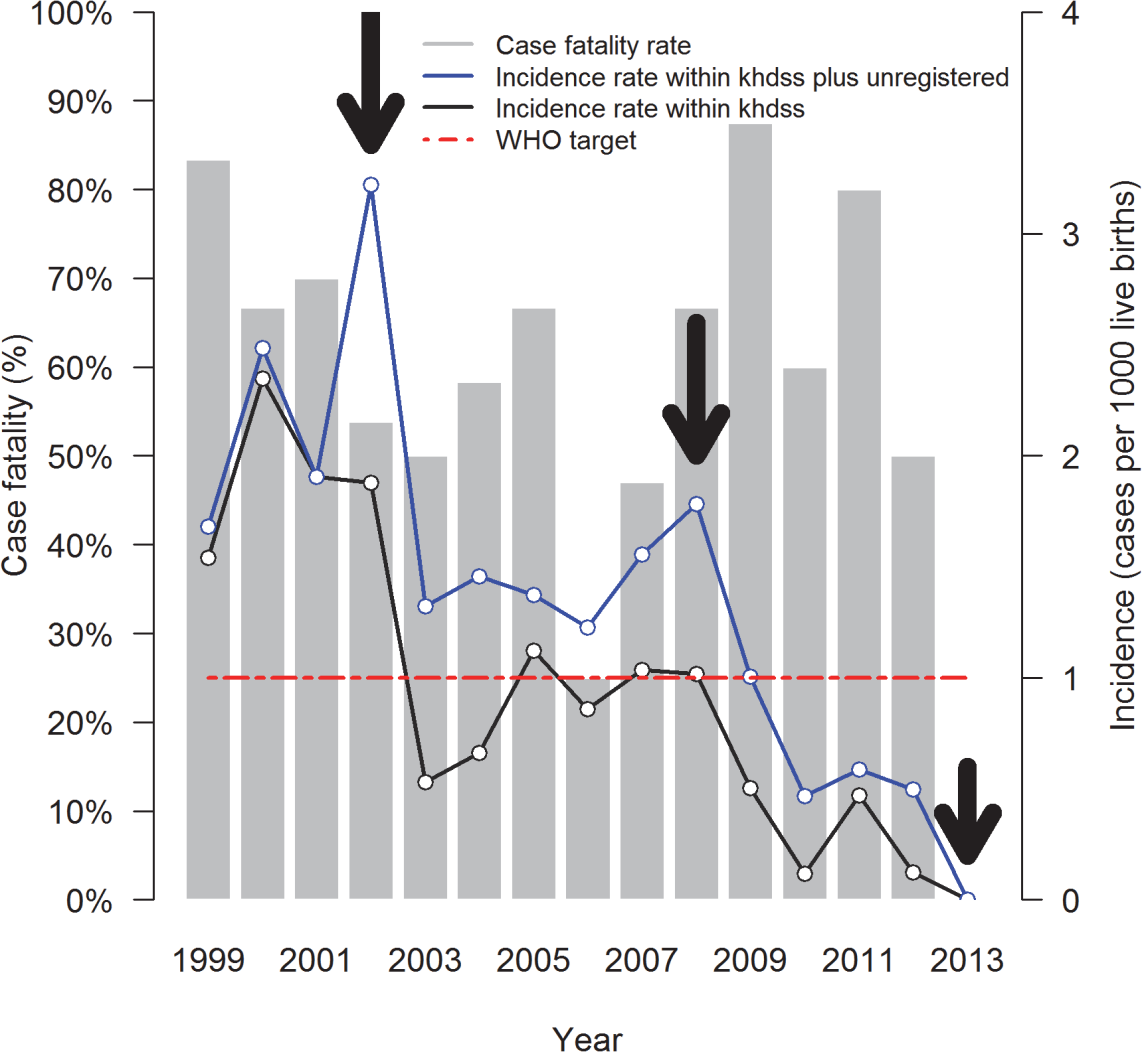
Incidence and case fatality of neonatal tetanus in Kilifi County Hospital. Incidence was computed as the number of Neonatal Tetanus (NT) cases within the KHDSS divided by the number of live births in that year. There were zero NT admissions in 2013 and therefore no deaths. NT campaigns started in 2002 in an effort by the Kenya Ministry of Health to abate NT. Major campaigns to women of child bearing age were carried out in 2002, 2008 and 2013 as shown by the arrows. The red dashed line is the WHO target of less than one NT cases per 1000 live births. The case fatality is the proportion of neonatal tetanus admissions discharged dead.

During the 15-year period, 191 neonates with NT were admitted to Kilifi County Hospital (KCH), of whom 118/189 (62.4%) were males. Thirty-four (17.8%) were admitted from outside the KHDSS while 50 (26.2%) were assumed to be unregistered because the location was unknown (S1 table). The remainder (107, 56.0%) were from within the KHDSS. The estimated incidence consistently declined from 2.3 to 0.0 per 1000 live births in between 1999 and 2013 (Incidence Rate Ratio: 0.85 (95% Confidence Interval (95% CI) 0.81–0.89), P<0.001) (Fig 1) (there were zero NT admissions in 2013), with reductions in incidence appearing after immunisation campaigns for women of childbearing age conducted by the Ministry of Health in 2002 and 2008 [21]. An additional campaign in 2006 targeted school-age girls [21], and its effect may therefore not be apparent for 5–10 years. A similar pattern of incidence is observed when unregistered cases are considered (IRR: 0.89 (95% CI 0.86–0.93), P<0.001) (Fig 1). Only in the years 2010, 2012 and 2013 were the upper limits of the 95% CI within the WHO target of less than one case per 1,000 live births.

### Characteristics of NT cases at admission

At admission 78/173 (45.1%) neonates with NT had inflamed cord stumps (Table 1). The median number of spasms experienced by the children every five minutes was 5 (Inter quartile range (IQR): 2–10). The median age and weight at admission were 7 days (IQR: 5–9) and 2.5kg (IQR: 2.3–2.8) respectively (S2 Table).

**Table 1:** Characteristics of children admitted with neonatal tetanus.

** **	**Survived**		**Died**	
** **	**N/mean**	**%/SD**	**N/mean**	**%/SD**
Overall	73	38%	118	62%
*Maternal features*				
Mothers not educated**	10/43	23.3%	33/85	38.8%
Mother not immunized by self-reporting	31/61	50.8	65/112	58.0
*Demographic features*				
Male	49/72	68.1%	69/117	59.0%
Age, days—median (IQR)^a^	10	8–13	6	5–7
*Clinical features*				
Heart rate (per minute)*	167.4	4.0	165.8	3.3
Respiratory rate (per minute)*	60.6	2.6	64.7	2.1
Inflamed umbilicus	22/66	33.3%	56/107	52.3%
Prostration	20/33	60.6%	50/60	83.3%
Number of Spasms*	4.15	3.1	6.9	5.5
NT score*	13.3	0.4	14.9	0.4
Low oxygen saturation (<95%)	16/66	24.2%	59/107	55.1%
Complication during delivery	1/70	1.4%	3/116	2.6%
*Laboratory Investigations*				
Hypoglycaemia (<2.5mmol/L)	7/33	21.2%	26/61	42.6%
Magnesium concentration (mmol/l)*	0.8	0.4	0.8	0.2
Sodium (mmol/l)*	145.1	8.3	143.5	6.4
Creatinine (μmol/l)*	99.2	70.9	94.1	45.2
White cell count (10^3^/μl)*	13.0	4.5	13.0	4.9
Haemoglobin (g/dl)*	15.1	2.6	15.6	3.2

* indicates that mean and standard deviation are shown since they are continuous variables.

**Mothers education was scored as not educated those who have never been to school and educated if they have been to school.

^a^ age in days at admission, median and interquartile ranges are shown, data available for 182 cases.

Most mothers 85/128 (66.4%) had never enrolled in school whilst 38/128 (29.7%) had primary level education, 5/128 (3.9%) secondary and none had post-secondary. Of 63 women for whom data were available, 41 (65%) were primigravidae. All mothers had a spontaneous vaginal delivery with only 4/186 (2.2%) having complications: one in hospital and three home births. There were only 4/187 (2.2%) hospital births. Of 158 cases for which data was recorded, 102 (65%) reported using nonsterile or harmful substance on the umbilicus (Table 1).

### Antitetanus antibodies

Of the 61 mothers tested, 11 (18%) had levels of antitetanus antibody above the limit of detection of our assay (~0.03 IU/ml). Approximately one third (4/11) of these mothers had antitetanus antibodies levels at or above ~0.2 IU/ml, the range in which ELISA levels correlate with *in vitro* neutralisation levels [6,7]. Sporadic cases of tetanus disease occur in individuals with >0.2 IU/ml [6,7,22,23], possibly because low affinity antibodies are measured, but protect poorly [7]. The four mothers with levels ≥0.2 IU/ml all had levels in the range of 0.2–1.0 IU/ml. The seven mothers with low levels (<0.2 IU/ml) all had levels in the range 0.03–0.1 IU/ml. Only one infant out of 47 (2%) with NT had detectable antitetanus antibodies in their blood (0.5 IU/ml), a child of a mother who had IgG level of 0.04 IU/ml. Thus, of the nine mothers with detectable antitetanus IgG and a paired child’s sample, eight had children with no detectable antitetanus IgG. We thus assessed the levels of maternal antitetanus IgG_1_. All four of the mothers with high antitetanus antibodies levels (≥0.1 IU/ml) had detectable IgG_1_, the subtype of IgG that most easily crosses the placenta to the fetus [24]. Of the mothers with low antitetanus antibodies levels (<0.1 IU/ml), 5/7 were tested for IgG_1_; four were negative and the fifth had low/equivocal levels.

The presence of antitetanus IgG only partially matched self-reported immunisation status: of the mothers lacking detectable antitetanus IgG, 30% (15/50) reported receiving at least one dose of tetanus vaccine. Of these 15, seven (14%) reported receiving only one or two doses, consistent with antibody levels decaying after insufficient immunisation. Among the mothers with detectable IgG, 73% (8/11) reported being immunised with <3 (5, 45%) or ≥3 (3, 27%) doses. Several possible reasons could explain the lack of detectable antitetanus IgG in the 15 reporting vaccination but did not have detectable antitetanus IgG levels: (i) they were mistaken about whether or not they were vaccinated against tetanus, (ii) they had immune responses that waned over time because of insufficient vaccination, (iii) they were given ineffective vaccines, perhaps with an interrupted cold chain [25,26] and/or (iv) they were non-responders.

### Factors associated with NT compared to hospital controls

We compared vital statistics of NT infants to those of 400 randomly selected neonatal admittees to KCH during the same time period. From the univariable logistic regression, NT was associated with home delivery, low blood oxygen saturation (<95%) and low weight at admission (<2.5kg), but was not associated with sex (Table 2). These associations remained significant in the multivariable logistic regression.

**Table 2:** Factors associated with neonatal tetanus cases compared with hospital controls*.

** **	**Cases**		**Controls**		**Univariable analysis**		**Multivariable analysis**	
** **	**N**	**%**	**N**	**%**	**OR(95%CI)**	**P value**	**OR(95%CI)**	**P value**
Home delivery	183/187	97.9	307/390	78.7	12.37 (4.46,34.30)	**<0.001**	14.67 (5.11,42.06)	**<0.001**
Male	118/189	62.4	235/399	58.9	1.16 (0.81,1.16)	0.414	-	-
Low weight at admission (<2.5kg)	35/191	18.3	29/400	7.3	2.87 (1.70,4.86)	**<0.001**	3.19 (1.76,5.08)	**<0.001**
Low oxygen saturation (<95%)	75/191	39.3	66/400	16.5	3.27 (2.21,4.84)	**<0.001**	3.29 (2.15,5.01)	**<0.001**

*Logistic regression was used to investigate the factors associated with death among the neonatal tetanus admittees.

### Outcomes and Risk factors on admission to hospital for death

Out of the 191 NT babies that were admitted, 73 (38.2%) were discharged alive. The case fatality was therefore 61.8%, and did not vary significantly over the 14 years (P = 0.536) (Fig 1). Age at admission, presence of inflamed umbilicus, prostration, number of spams per 5 minutes, NT score and presence of hypoglycaemia were all associated were all significantly associated with death in the univariable analysis (Table 3). Eight variables with P<0.20 in the univariable analysis were included in the multivariable logistic model to identify the factors which were independently associated with death. Presence of maternal antitetanus IgG was not associated with risk of neonatal death: (62% (31/50) vs 55% (6/11), nor did reported antitetanus immunisation (Table 3). This suggests that antibody in the infant at levels below the limit of detection of our assay transferred from the mother did not play a major role in protecting children from death. Frequency of spasms was not included in the multivariable model because it was highly correlated with and partially derived from NT score. Age in days at admission (OR = 0.73, 95% CI 0.65–0.83, P = 0.001) was the only factor found to be independently associated with mortality (Table 3), with younger neonates having the highest odds for death.

**Table 3:** Univariable and multivariable analysis of the factors associated with death in children with neonatal tetanus*.

** **	**Univariable analysis**		**Multivariable analysis**	
Overall	**OR (95%CI)**	**P value**	**OR (95%CI)**	**P value**
*Maternal features*				
Mothers not educated	2.09 (0.91–4.81)	0.081	0.73 (0.18–2.97)	0.662
Mother not immunized by self-reporting	1.34 (0.72–2.50)	0.362	-	-
*Demographic features*				
Male	0.67 (0.36–1.25)	0.212	-	-
Age in days	0.73 (0.65–0.83)	**<0.001**	0.61 (0.46–0.82)	**0.001**
*Clinical features*				
Heart rate (per minute)	1.00 (0.99–1.01)	0.759	-	-
Respiratory rate (per minute)	1.01 (0.99–1.03)	0.217	-	-
Inflamed umbilicus	2.20 (1.16–4.15)	**0.016**	1.57 (0.45–5.41)	0.476
Prostration	3.25 (1.23–8.61)	**0.018**	1.30 (0.33–5.16)	0.713
Number of Spasms	1.15 (1.04–1.26)	**0.007**	-	-
NT score	1.15 (1.04–1.27)	**0.006**	1.11 (0.92–1.34)	0.284
Low oxygen saturation (<95%)	3.56 (1.84–6.90)	**<0.001**	1.40 (0.35–5.58)	0.876
Complication during delivery	1.83 (0.19–8.0)	0.603	-	-
*Laboratory Investigations*				
Hypoglycaemia (<2.5mmol/L)	2.76 (1.04–7.33)	**0.042**	4.80 (0.87–26.57)	0.072
Magnesium (mmol/l)	2.16 (0.01–506.19)	0.783	-	-
Sodium (mmol/l)	0.97 (0.91–1.03)	0.317	-	-
Creatinine (μmol/l)	1.00 (0.99–1.00)	0.680	-	-
White cell count (10^3^/μl)	1.00 (0.91–1.09)	0.990	-	-
Haemoglobin (g/dl)	1.06 (0.92–1.23)	0.414	-	-

*In the multivariable logistic model, eight variables with a p-value<0.20 were considered in identifying the independent risk factors for death. Frequency of spasms was not included in the multivariable model as it was highly correlated with neonatal tetanus score (NT score).

## Discussion

This study examined the incidence and outcomes of NT based on hospital admissions in a carefully monitored health and demographic surveillance site in rural Kenya, over a long period of time (15 years). There was a marked decline in incidence over this period (1999–2013) with the point estimates of incidence for the last five years (2009–2013) being <1 case per 1000 live births per year. The proportion of male cases was significantly higher than the expected 50% **(χ** ^2^ = 5.95, P = 0.015), as reported in other studies [21,27,28], but not higher than the proportion of males in our neonatal hospital controls from KCH (Table 2). A large population study suggests that males have a higher risk of severe bacterial infections in the neonatal period [29], possibly due to greater biological susceptibility.

Among NT cases, younger infant age at admission was the only factor independently associated with mortality. Earlier presentation with disease may be due to higher inocula of *Clostridium tetan*i bacteria, and/or younger infants may be physiologically at higher risk of death [27,30].

### Changes in incidence over time

Reductions in incidence of NT over time can be ascribed to several factors. First, although the majority of births in sub-Saharan Africa still occur at home, the numbers of hospital deliveries (where births are attended by skilled attendants) are increasing. From the 2008–2009 Kenyan Demographic and Health survey, it was estimated that 44% of the total Kenyan births occur in the hospital [31]. In Kilifi, about 40% of births are estimated to occur at home [21]. However, in Kilifi, over a 19-year period, (1989 to 2008), hospital births have almost doubled [15], while the population rose only by 32% in the KHDSS from 2001 to 2011 [16], suggesting that the proportion of hospital births is increasing. Deliveries in health facilities are more likely to use anti-sepsis, decreasing the likelihood of NT. In support of this idea, only 2% of NT cases were born at a health care facility. In a prior study, home deliveries were also associated with NT [32]. These associations with home deliveries may be related to the use of unsterilized instruments to cut the cord, coupled with applying non-sterile after-birth substances to cauterize the unhealed cord stump [28,33,34]. The use of unclean delivery and cord care practices may be related to low maternal education levels [35], as suggested by the apparently high rate of mothers with low levels of education. A review done in Kilifi, Kenya between 2004 and 2007 found an increasing incidence of NT cases (0.6 to 1 per 1000 live births) [21]. However, that study did not attempt to assess the statistical significance of this trend, and that small trend runs counter to the overall trend in our larger data set.

Importantly, increased immunisation coverage of women of child-bearing age received TT5, likely contributed. Decreases in incidence follow immunisation campaigns from the Kenya Ministry of Health in 2002, 2008 and 2013 which targeted women of child bearing age. 2006 focussed on school going children (<15 years) and the effect would be expected to be apparent few years later. The sustained drop in incidence from 2009 may result from reaching a threshold of better immunisation coverage and/or from external factors such as increasing ease of transportation and urbanisation leading to changes in lifestyle, health-seeking behaviour and likelihood of delivering in a health care facility [36]. Anecdotal reports indicate that the early antitetanus immunisation campaigns targeting women of childbearing age suffered from misconceptions that family planning drugs were to be included with the immunisations and that these misconceptions resulted in low uptake. This included the 2002 tetanus immunisation campaign in the Coastal region, which includes Kilifi County [37]. We were aware at the time of similar anecdotes from local Ministry of Health workers participating in that campaign in Kilifi County. These misconceptions may have lessened over time; if so, the later campaigns may have been more successful.

### Antitetanus antibodies

Most mothers of neonates with tetanus we tested (50/61, 82%) did not have detectable levels of antitetanus antibody in their blood. Among the minority of women that did have measurable antitetanus antibody, all but one failed to transfer antibody that was detectable in the admission sample of the neonatal case. One infant had detectable antitetanus antibody above 0.1 IU/ml, which is usually associated with protection. In addition, there are reports in the literature of one or two dozen cases of patients with tetanus who had >0.1 IU/ml [6,23], suggesting that antitetanus antibodies, at least as measured by ELISA do not always protect from tetanus. It is thought that ELISA may also detect antibodies that are not necessarily protective [6].

Of the nine mothers with detectable antitetanus antibody and a paired child’s blood sample, eight failed to transfer antitetanus antibody at a level detectable on admission. This proportion of failures appears unusually high. It seems clear that by selecting infants with NT, we may have selected for those who did not receive antitetanus antibodies from their mothers, and the proportion of failures is presumably lower in the general population. The mechanism of this failure is unclear. We measured antitetanus IgG_1_ levels because IgG_1_ is the subtype of antibody most readily transferred across the placenta [24]. Antitetanus IgG_1_ was detected in all 4 mothers with high antitetanus antibody levels that did not transfer detectable antitetanus antibody to her infant, yet in none of the mothers with low antitetanus antibody levels, including the mother of the one child found with antitetanus antibodies. We presume that this reflects the detection limit of our IgG_1_ assay, and suggests that failure to produce IgG_1_ is not a mechanism of failure to transfer antibody to the foetus in this population. The transfer of antibody to the foetus during pregnancy may be impaired by placental malaria [38–40] or HIV infection of the mother [41–43]. We were not able to assess mothers for either HIV status or for placental malaria infection. However, it is plausible that either of these infections may have influenced maternal transfer of antibody during pregnancy.

### Case fatality and risk factors

High case fatality for NT is common [29], especially in resource-poor areas where there are no facilities for ventilation, poor glucose monitoring and intravenous antitetanus immunoglobulin is not available. In contrast in high income regions, where cases are extremely rare, survival has improved greatly due to intensive care facilities [28,44]. Case fatality in this study is lower than historical data for Kilifi [21,45], possibly due to improved care due to the high dependency unit and the proximity of a research facility with more skilled staff.

Those who died were significantly younger than those who survived. We and others [27,30] speculate that the shorter incubation period may be related to larger initial innocula with *Clostridium tetan*i bacteria, and/or younger infants may be at higher risk of death. There is little independent confirmation for this conclusion.

### Strengths and Limitations

This study analyses a relatively large number of NT cases (191) over a long period of time (15 years) in Kilifi County in coastal Kenya, a region with historically high incidence of neonatal tetanus. Although this study used reliable live births estimates [16], the incidence may have been underestimated because it only included NT cases admitted to the hospital.

## Conclusions

NT incidence has dropped over the last 15 years in this rural region of Kenya. This achievement is contemporaneous with vaccination campaigns and with an apparent increase in the rate at which delivering mothers turn to health care facilities for birth. These gains should be built on. If these changes are sustained and apply to the rest of the country, Kenya is well on its way to reaching the WHO elimination goal. Global elimination of this fatal disease is achievable, and should be expedited, as we seek to reduce the substantial burden of neonatal mortality worldwide.

## Supporting Information

S1 Table Distribution of cases of neonatal tetanus admittees with the place of residence. This table shows the distribution of admissions with neonatal tetanus to Kilifi County Hospital over a 15-year period. Some of the cases were unregistered and it was therefore not possible to tell whether they case from the covered by the Kilifi Health and Demographic Surveillance System (KHDSS). The live births for the period 1999–2003 were predicted from a simple linear regression with number of live births for the rest of the years as the outcome variable and year as the independent variable.
(DOCX)

S2 Table Tabulation of discharge status by age among the neonatal tetanus admittees. (DOCX)
