# Supplementary material for: Incidence and Risk Factors for Neonatal Tetanus in Admissions to Kilifi County Hospital, Kenya
Source: PLoS One. 2015 Apr 7;10(4):e0122606. doi: 10.1371/journal.pone.0122606 (PMC4388671; doi:10.1371/journal.pone.0122606)
Supplement: S1 Table — This table shows the distribution of admissions with neonatal tetanus to Kilifi County Hospital over a 15-year period. Some of the cases were unregistered and it was therefore not possible to tell whether they case from the covered by the Kilifi Health and Demographic Surveillance System (KHDSS). The live births for the period 1999–2003 were predicted from a simple linear regression with number of live births for the rest of the years as the outcome variable and year as the independent variable. (DOCX) [file pone.0122606.s001.docx]

| Year | Neonatal tetanus cases | | | | | Live births within KHDSS |
| --- | --- | --- | --- | --- | --- | --- |
|  | Total | unregistered | within KHDSS | Outside KHDSS | Died |  |
| 1999 | 18 | 1 | 11 | 6 | 15 | 7134 |
| 2000 | 24 | 1 | 17 | 6 | 16 | 7239 |
| 2001 | 20 | 0 | 14 | 6 | 14 | 7344 |
| 2002 | 26 | 10 | 14 | 2 | 14 | 7449 |
| 2003 | 10 | 6 | 4 | 0 | 5 | 7554 |
| 2004 | 12 | 6 | 5 | 1 | 7 | 7542 |
| 2005 | 15 | 2 | 9 | 4 | 10 | 8009 |
| 2006 | 12 | 3 | 7 | 2 | 3 | 8147 |
| 2007 | 17 | 4 | 8 | 5 | 8 | 7714 |
| 2008 | 15 | 6 | 8 | 1 | 10 | 7852 |
| 2009 | 8 | 4 | 4 | 0 | 7 | 7943 |
| 2010 | 5 | 3 | 1 | 1 | 3 | 8528 |
| 2011 | 5 | 1 | 4 | 0 | 4 | 8504 |
| 2012 | 4 | 3 | 1 | 0 | 2 | 8013 |
| 2013 | 0 | 0 | 0 | 0 | 0 | 8118 |
