# Supplementary material for: Incidence and Risk Factors for Neonatal Tetanus in Admissions to Kilifi County Hospital, Kenya
Source: PLoS One. 2015 Apr 7;10(4):e0122606. doi: 10.1371/journal.pone.0122606 (PMC4388671; doi:10.1371/journal.pone.0122606)
Supplement: S2 Table — (DOCX) [file pone.0122606.s002.docx]

| Age in days at admission | Survived | Died | Total |
| --- | --- | --- | --- |
| 0 | 0 | 2 | 2 |
| 1 | 0 | 1 | 1 |
| 3 | 0 | 4 | 4 |
| 4 | 0 | 4 | 4 |
| 5 | 3 | 32 | 35 |
| 6 | 2 | 25 | 27 |
| 7 | 8 | 19 | 27 |
| 8 | 9 | 13 | 22 |
| 9 | 10 | 7 | 17 |
| 10 | 8 | 2 | 10 |
| 11 | 5 | 0 | 5 |
| 12 | 5 | 0 | 5 |
| 13 | 3 | 0 | 3 |
| 14 | 4 | 0 | 4 |
| 15 | 2 | 1 | 3 |
| 16 | 1 | 1 | 2 |
| 17 | 2 | 1 | 3 |
| 19 | 0 | 1 | 1 |
| 20 | 1 | 0 | 1 |
| 22 | 1 | 0 | 1 |
| 23 | 0 | 2 | 2 |
| 28 | 2 | 0 | 2 |
| 29 | 1 | 0 | 1 |
| Total | 67 | 115 | 182 |
